# P02.56. A naturopathic treatment protocol for acute otitis media: a report of twenty-four cases

**DOI:** 10.1186/1472-6882-12-S1-P112

**Published:** 2012-06-12

**Authors:** R Barrett

**Affiliations:** 1 Helfgott Research Institute, National College of Natural Medicine, Portland, USA

## Purpose

To describe a case series of children treated with a naturopathic protocol for acute otitis media (AOM). Acute otitis media places a large burden on pediatric populations. Antibiotics provide modest benefits; however, these benefits must be weighed against the risk of adverse events and antibiotic resistance. Current American Academy of Pediatrics guidelines includes “watchful waiting”: withholding antibiotics and providing analgesics and supportive care in selected patients.

## Methods

A retrospective case series was compiled by searching the electronic records from 2010-2011 from pediatrics teaching rotations at two naturopathic teaching clinics. Thirty cases were identified. Stringent criteria were applied to increase diagnostic accuracy, as viral respiratory infections are often misclassified as AOM. Inclusion criteria were: (1) acute onset of otalgia (2) tympanic membrane (TM) cloudiness or redness (3) Impaired TM mobility (4) TM bulging or fullness. Twenty-four children between 2 and 7 years-of-age met the inclusion criteria (average age 37 months, 17 boy: 7 girls). Cases were treated with a whole practice naturopathic treatment, including a botanical formula, nutritional supplementation, low-antigenic diet and hydrotherapy. Seventy-five percent received an acute homeopathic prescription and 54% received botanical analgesic eardrops. Primary outcomes: pain duration, assessed at 24 hours and days 2-7. Secondary outcomes: treatment failure, complications of AOM, and adverse events. Tympanometry was performed in 9 children at approximately 1 month to assess for residual effusion and hearing impairment.

## Results

The number of children with pain at 24 hours was 7 of the 24 (31%). No children had pain by days 2-7. Adverse events: One patient discontinued the eardrops due to the garlic odor. There were no perforations or suppurative complications. Among the group that returned for tympanometric assessment, 6 of 7 (86%) had normal (Type A) tympanograms.

## Conclusion

Naturopathic protocols may be a valuable adjunct in patients selected for watchful waiting for management of AOM.

